# Organic Acid Exposure Enhances Virulence in Some *Listeria monocytogenes* Strains Using the *Galleria mellonella* Infection Model

**DOI:** 10.3389/fmicb.2021.675241

**Published:** 2021-07-06

**Authors:** Minghao Li, Charles E. Carpenter, Jeff R. Broadbent

**Affiliations:** Department of Nutrition, Dietetics and Food Sciences, Utah State University, Logan, UT, United States

**Keywords:** organic acid, acid resistance, bile resistance, virulence, gene expression, *Listeria monocytogenes*, *Galleria mellonella*

## Abstract

Prior research has suggested that the use of organic acids in the food industry may unintentionally enhance pathogenicity of *Listeria monocytogenes* strain N1-227 and R2-499. This study explored the connection between habituation to L-lactic acid or acetic acid and virulence in *L. monocytogenes* strains N1-227 and R2-499 using selected gene expression analysis and the *in vivo Galleria mellonella* wax worm model for infection. Expression of transcription factors (*sigB* and *prfA*) and genes related to acid resistance (*gadD2, gadD3*, and *arcA*) and bile resistance (*bsh* and *bilE*) or to virulence (*inlA, inlB, hly, plcA, plcB, uhpT*, and *actA*) was investigated by quantitative real-time PCR (qRT-PCR), while *in vivo* virulence was assessed by following the lethal time to 50% population mortality (LT_50_) of *G. mellonella* larvae after injection of untreated and habituated *L. monocytogenes.* Twenty minutes of habituation to the organic acids at pH 6.0 significantly increased expression of key acid and bile stress response genes in both strains, while expression of virulence genes was strain-dependent. The expression of transcription factor *sigB* was strain-dependent and there was no significant change in the expression of transcription factor *prfA* in both strains. Habituation to acid increased virulence of both strains as evidenced by decreased LT_50_ of *G. mellonella* larvae injected with *Listeria* habituated to either acid. In summary, habituation of both *L. monocytogenes* strains to organic acids up-regulated expression of several stress and virulence genes and concurrently increased virulence as measured using the *G. mellonella* model.

## Introduction

The genus *Listeria* is comprised of Gram-positive, non-spore-forming, rod-shaped, facultative anaerobic bacteria which can be found ubiquitously in the environment (Mélanie et al., 2006; Gahan and Hill, 2014; Lani and Hassan, 2016). Among *Listeria* species, only *L. monocytogenes* and *L. ivanovii* are pathogenic (Robinson and Batt, 1999); *L. ivanovii* primarily infects animals while *L. monocytogenes* shows pathogenicity toward both humans and animals (Liu, 2006). During food production, *L. monocytogenes* can experience several stresses such as low pH and high salt. The ability of *Listeria* to adapt to these adverse conditions plays a crucial role in food contamination and food-borne infection (Lani and Hassan, 2016).

In response to stress, *L. monocytogenes* may induce an acid tolerance response and other stress responses mechanisms that allow it to overcome these hurdles (Glass et al., 1995; Silva et al., 2012; Melo et al., 2015). *L. monocytogenes* is able to utilize a variety of regulators (over 100 different transcriptional regulators have been identified) to survive and grow in different environments (Glaser et al., 2001; Gaballa et al., 2019). Among those regulators, the alternative sigma factor B (σ^*B*^) and the listeriolysin positive regulatory factor A (PrfA) are two essential transcriptional regulators for stress response and for host infection.

σ^*B*^, encoded by *sigB*, is a general stress responsive transcription sigma factor in *L. monocytogenes* and many other Gram-positive bacteria (Kazmierczak et al., 2005; Chaturongakul et al., 2008). In *L. monocytogenes*, σ^*B*^ regulates numerous genes that are associated with acid, bile and other physiological stressors (Sue et al., 2004; Zhang et al., 2011; Smith et al., 2012; Melo et al., 2015). The acid stress response systems in *L. monocytogenes* include the glutamate decarboxylase (GAD) system and an arginine deiminase (ADI) system. The GAD system, which involves genes encoding three glutamate decarboxylase enzymes (*gadD1, gadD2* and *gadD3*) and two gamma aminobutyric acid (GABA) antiporters (*gadT1* and *gadT2*), plays a significant role in pH homeostasis in *L. monocytogenes* (Cotter et al., 2001; Melo et al., 2015). Expression of the GAD system results in the decarboxylation of glutamate into γ-aminobutyrate with consumption of intracellular protons (Cotter et al., 2001; Karatzas et al., 2012). Additionally, the arginine deiminase (ADI) system also contributes to the stabilization of the bacterial cytoplasmic pH (Melo et al., 2015). The ADI pathway involves the enzymes arginine deiminase, ornithine carbamoyltransferase and carbamate kinase, which are encoded by *arcA*, *arcB*, and *arcD*, respectively (Melo et al., 2015). With respect to bile resistance, one of the most important mechanisms in *L. monocytogenes* involves the ability to detoxify individual conjugated bile acid through bile salt hydrolase (BSH) (Dussurget et al., 2002; Begley et al., 2005). Another novel bile resistance system in *L. monocytogenes* is the bile exclusion system (BilE), which acts to exclude bile from bacterial cells (Sleator et al., 2005).

The listeriolysin positive regulatory factor A (PrfA), encoded by *prfA*, is a bacterial transcription factor that controls and coordinates the expression of key virulence genes in *L. monocytogenes* associated with cell invasion and the intracellular infection cycle (Kazmierczak et al., 2006; Scortti et al., 2007; de las Heras et al., 2011). Cell invasion is mediated by two surface proteins, internalin A and B (InlA and InlB); after entering the cell, *L. monocytogenes* are entrapped in a phagocytic vacuole from which they escape by lysing the membrane of the vacuole through the combined actions of the pore-forming toxin listeriolysin O (LLO, encoded by *hly*) and two phospholipases, PlcA and PlcB (Mélanie et al., 2006). Multiplication and invasion within host cells can then occur with the involvement of the permease UhpT (a hexose phosphate transporter) and the surface protein ActA (propel bacteria through the cytoplasm) (Chico-Calero et al., 2002; Mélanie et al., 2006; Cossart and Toledo-Arana, 2008).

Acid stress resistance has been well studied and observed in various microorganisms such as *Escherichia coli* (Goodson and Rowbury, 1989) and *Salmonella* (Foster and Hall, 1990). Prior research by our group has suggested that the use of organic acids in the food industry may unintentionally enhance virulence of some *L. monocytogenes* strains (Zhang et al., 2014). Those results showed that habituation of two *L. monocytogenes* strains, N1-227 and R2-499, to organic acid under mildly acidic conditions (pH = 6.0) induced acid and bile resistance, which indicated these treatments could promote virulence by enhancing survival during passage through the gastrointestinal tract (Zhang et al., 2014). It also suggested the increased acid and bile resistance was specifically due to organic acid exposure rather than a decrease in environmental pH (Carpenter and Broadbent, 2009; Zhang et al., 2014). Similar responses were not observed in that study with other pathogenic strains of *L. monocytogenes* (Zhang et al., 2014), so R2-499 and N1-227 were selected for further study to explore the genetic basis for inducible acid and bile resistance, and to determine if it affected virulence in an *in vivo* model.

Virulence of *Listeria* spp. is frequently assessed using a murine model (Lecuit, 2007). However, this model has limitations for studying human pathogenicity of *L. monocytogenes* because the interaction between InlA and mouse E-cadherin (identified as InlA receptor in human) is poor, which makes *L. monocytogenes* entry into epithelial cells less efficient (Mengaud et al., 1996; Lecuit et al., 1999). The larvae of *Galleria mellonella* have also been used as a model for *L. monocytogenes* virulence (Joyce and Gahan, 2010; Mukherjee et al., 2010, 2013; Banville et al., 2012; Ramarao et al., 2012; Schrama et al., 2013). Compared to the mammalian model and other alternative models, the *G. mellonella* model offers several significant advantages, including structural and functional similarities with the mammalian immune system (Hoffmann et al., 1999; Strand, 2008). Additionally, the infection process can be performed over a range of temperatures (from 15°C to above 37°C), which enables use of the *G. mellonella* model to study the virulence of *L. monocytogenes* human pathogens at 37°C (Jones et al., 2010; Rejasse et al., 2012).

To better understand the molecular basis and potential consequences of induced acid and bile resistance in organic acid habituated strains, we used quantitative real time polymerase chain reaction (qRT-PCR) to measure the expression of key transcription factors and some of their target genes related to acid or bile resistance or virulence in *L. monocytogenes* strains N1-227 and R2-499 after habituation to lactic acid or acetic acid at pH 6.0. Additionally, the *G. mellonella* infection model was used to analyze the *in vivo* virulence of control and acid habituated *L. monocytogenes* strains.

## Materials and Methods

### Bacterial Strains and Growth Conditions

Original cultures (Table 1) were stored as frozen stocks at −80°C in tryptic soy broth (TSB, pH 7.4; Becton, Dickinson and Company, Sparks, MD) supplemented with 20% v/v glycerol. Prior to use, cultures were first propagated on tryptic soy agar (TSA; Becton, Dickinson and Company, Sparks, MD) plate and incubated at 37°C for 24 h. A single colony from the TSA plate was transferred into TSB and incubated overnight at 37°C with shaking (220 rpm).

**TABLE 1:** Listeria monocytogenes strains used in this study.

**Strain**	**Ribotype**	**Lineage**	**Serotype**	**Source**
FSL R2-499	DUP-1053A	II	1/2a	Human isolate associated with the US outbreak linked to sliced turkey, 2000
FSL N1-227	DUP-1044A	I	4b	Food isolate associated with the US outbreak, 1998–1999

### RNA Isolation

Overnight cultures of each strain were harvested by centrifugation (2,500 × g for 10 min; Sorvall RT1, Thermo Scientific, Germany) at 4°C, and then diluted to an optical density at 600 nm (OD_600_) of 0.03 in TSB. Cells were acid habituated as described by Zhang et al. (2014). A 1% inoculum (v/v) of diluted overnight cultures was transferred into 50 mL of standard TSB (pH 7.4) and incubated at 37°C for 4 h with shaking (220 rpm) to reach mid-log phase as determined by Zhang et al. (2014). The cultures were collected by centrifugation (2,500 × g for 10 min) at 4 °C and then suspended in 50 mL of either standard TSB (pH 7.4, baseline control) or TSB without dextrose (pH 6.0 adjusted with HCl, Becton, Dickinson and Company, Sparks, MD) containing 0 (pH control) or 4.75 mM of either L-lactic acid (Sigma Chemicals, St. Louis, MO) or acetic acid (Johnson Matthey Company, Ward Hill, MA). The cultures were incubated at 37°C for 20 min with shaking (220 rpm). After incubation, 100 mL of RNAprotect Bacteria Reagent (Qiagen, Inc., Valencia, CA) was added to each sample. Cells were incubated at room temperature for 10 min then collected by centrifugation (9,500 × g for 10 min). The supernatant was discarded and cell pellets were suspended in 900 μL of lysozyme solution (Sigma-Aldrich, 20 mg/mL in Tris-EDTA buffer) that contained 20 units of mutanolysin (Sigma-Aldrich). Samples were incubated for 30 min at 37°C on a shaker incubator at 220 rpm, then 20 μL of proteinase K (Omega Bio-Tek Inc., Norcross, GA) (20 mg/mL) was added and the samples were returned to the shaker/incubator for 30 min. Total RNA was isolated using an Aurum total RNA mini kit (Bio-Rad, Hercules, CA) following the vendor’s recommended procedures. Residual DNA was removed using The Ambion^®^ DNA-free^TM^ DNase Treatment and Removal Reagents. RNA samples were then purified using the GeneJET RNA Cleanup and concentration Micro Kit PCR purification kit (Thermo Fisher Scientific, Lithuania). The amount and quality of the RNA were measured using a NanoDrop Spectrophotometer (Thermo Fisher Scientific, United States) and TapeStation System (Agilent, Santa Clara, CA), respectively.

### cDNA Synthesis and Real Time Quantitative PCR (qPCR)

cDNA was synthesized from total RNA using random primers (Invitrogen, Carlsbad, CA) and SuperScript II reverse transcriptase (Invitrogen). The qPCR was carried out using cDNA as template in an Opticon II thermal cycler (MJ Research, Reno, NV) using HotStart-IT^TM^ SYBR Green qPCR Master Mix with UDG kit (Affymetrix, Inc.). Each reaction was performed in triplicate and the relative gene expression of targeted genes was calculated by the Pfaffl Method and normalized by the baseline control (Pfaffl, 2001). The primers used in this study are listed in Table 2 and *rpoB* was used as a housekeeping gene to normalize the gene expression data (Bookout and Mangelsdorf, 2003; Tasara and Stephan, 2007). The amplification efficiency for each primer was tested by plotting the cycle threshold (Ct) value with different template concentrations and fitting the data to a regression line (Bookout and Mangelsdorf, 2003; Ruijter et al., 2009). The amplification efficiency for all the primers reached 90% or above (Li, 2020).

**TABLE 2:** Primers used in this study.

**Protein**	**Function**	**Gene**		**Sequence (5′– > 3′)**
General stress-responsive sigma factor B	Required for the expression of *L. monocytogenes* stress response factors	*sigB*	F	TGTTGGTGGTACGGATGATGG
			R	ACCCGTTTCTTTTTGACTGCG
Arginine deiminase	Catalyze L-arginine to L-citrulline	*arcA*	F	GCGTGATTGCGGAGGTTTTG
			R	CCCCATCATTCCACTGCTCT
Glutamate decarboxylase β	Convert glutamate to GABA	*gadD2*	F	ATCGATATGCGCGTTGTTCCA
			R	ATACCGAGGATGCCGACCACA
Glutamate decarboxylase γ	Convert glutamate to GABA	*gadD3*	F	TTCCGCATTGTTACGCCAG
			R	TCTTACTTGGGGACTTCGAC
Bile salt hydrolase	Detoxify conjugated bile acid	*Bsh*	F	TTTGTTGTTCCACCGAGCCTA
			R	GGGCGGAATTGGCTTACCTG
Bile exclusion protein	Exclude bile from cell	*bilE*	F	CATCAACGGAGCCTGTCGAA
			R	TCCAGATGACGCGCTAAGAA
Positive regulatory factor A	Required for the expression of *L. monocytogenes* virulence factors	*prfA*	F	CGATGCCACTTGAATATCCT
			R	CTTGGCTCTATTTGCGGTCA
Internalin A	Host cell invasion	*inlA*	F	CTATACCTTTAGCCAACCTGT
			R	GGTTGTTTCTTTGCCGTCCAC
Internalin B	Host cell invasion	*inlB*	F	CTGGACTAAAGCGGAAAACCTT
			R	TCCAGACGCATTTCTCACTCTT
Listeriolysin O	Phagosome lysis	*hly*	F	ATGCAATTTCGAGCCTAACC
			R	ACGTTTTACAGGGAGAACATC
Phosphatidylinositol-specific phospholipase C	Phagosome lysis	*plcA*	F	ACCGTATTCCTGCTTCTAGTT
			R	ACACAACAAACCTAGCAGCG
Phosphatidylcholine phospholipase C	Phagosome lysis	*plcB*	F	TAGTCAACCTATGCACGCCAA
			R	TTTGCTACCATGTCTTCCGTT
Actin assembly-inducing ptotein	Stimulates actin-based intracellular bacterial motility	*actA*	F	TTATGCGTGCGATGATGGTG
			R	TTCTTCCCATTCATCTGTGT
Hexose phosphate transporter	Intracellular bacterial growth	*uhpT*	F	TTCAGCACCACAGAACTAGG
			R	GCATTTCTTCCATCCACGAC
RNA polymerase beta subunit	Housekeeping gene	*rpoB*	F	CTCTAGTAACGCAACAACCTC

### *Galleria mellonella* Wax Worm Model

**FIGURE 1:**
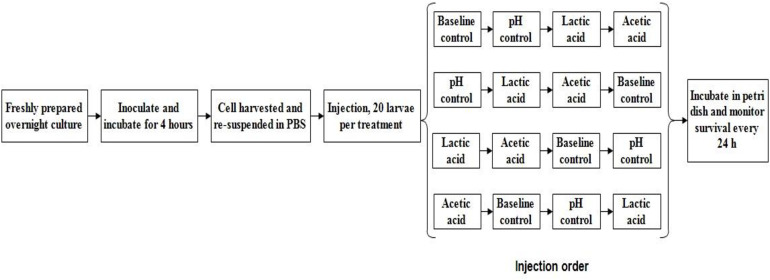
*Galleria mellonella* schematic experimental design of one biological repetition.

The *in vivo* virulence of *L. monocytogenes* strains was determined using the *Galleria mellonella* wax worm model described by Ramarao et al. (2012). A 1% inoculum (v/v) of freshly prepared *L. monocytogenes* cells was transferred into 50 mL of either standard TSB (pH 7.4, baseline control) or TSB without dextrose (pH 6.0 with HCl) containing 0 (pH control) or 4.75 mM of either L-lactic acid or acetic acid and incubated at 37°C for 4 h with shaking (220 rpm). The mid-log phase cultures were collected by centrifugation (2,500 × g for 10 min) at 4°C. The bacterial cells were then re-suspended with sterile PBS solution (pH 7.4) and diluted to an optical density at 600 nm (OD_600_) of 0.25. Ten microliters of 10^8^ cfu/mL *L. monocytogenes*, either control or acid habituated, was injected into the haemocoel of the wax worms using an automated syringe pump (KDS 100, KD Scientific; 20 larvae per treatment; see Figure 1 for schematic experimental design and injection order. Injection was done in two biological repetition). A PBS-only control injection was also included. The larvae were placed in petri dish (5 per dish) and incubated at 37°C. Larvae survival was evaluated every 24 h for 5 days after injection. The larvae were considered dead when they showed no movement in response to finger touch. Lethal times until 50% population mortality (LT_50_) for each treatment were then determined by Probit analysis (Bliss, 1934, 1935).

### Enumeration of *Listeria monocytogenes* in *Galleria mellonella* Wax Worms

*L. monocytogenes* in *G. mellonella* larvae was enumerated at 5, 10, 15, and 20 h after injection. At each time point, 5 larvae were collected and homogenized in 9 mL of sterile peptone physiological solution (PPS) in a stomacher. Serial dilutions were made by pipetting 1 mL of diluted sample into 9 mL PPS, then 100 μL of diluted samples was spread on Palcam agar (*L. monocytogenes* selective media; Oxoid Limited, Hampshire, United Kingdom). Plates were incubated at 37°C for 48 h then *L. monocytogenes* colonies were enumerated. Microbiological count data were expressed as log_10_ of colony-forming units per larvae.

### Statistical Analysis

The data collected in this study (relative expression ratio of target genes compared with reference genes in three biological repetitions, the survival rate of *G. mellonella* larvae and the enumeration of *L. monocytogenes* in *Galleria mellonella* wax worm in two biological repetitions) were continuous outcome variables for every categorical treatment variable (acidification treatments of *L. monocytogenes*). Significant differences in each outcome between treatments were assessed using one-way analysis of variance (ANOVA) followed by Tukey’s test to compare means of the gene expression outcome variables between treatments. Differences were considered significant at *P* < 0.05.

## Results

### Influence of Acid Habituation on Expression of Acid and Bile Stress Response Genes

**FIGURE 2:**
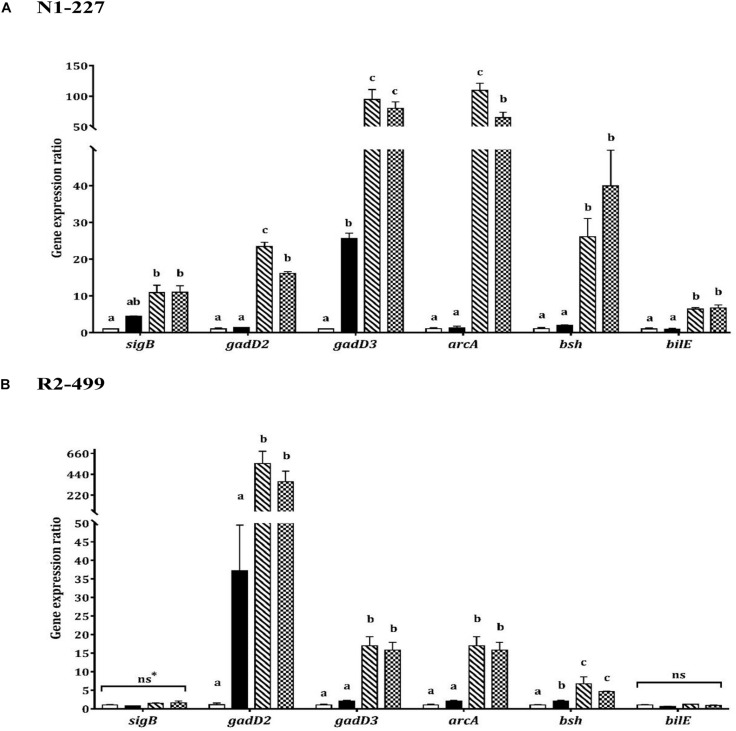
Relative gene expression of acid and bile stress response related genes in 20 min habituated *Listeria monocytogenes* strains **(A)** N1-227 and **(B)** R2-499 cells in comparison with non-habituated cells (baseline control, TSB pH 7.4, 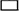). Habituated treatments include as follows: TSB pH 6.0 (pH control, 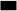), TSB at pH 6.0 with 4.75 mM of acetic acid (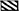) and TSB at pH 6.0 with 4.75 mM of L-lactic acid (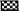). Error bars represent standard error of mean for two biological trials with three replicates for each trial. Different letters indicate that treatments are significantly different (*p* < 0.05) as determined by one-way ANOVA with Tukey’s *post-hoc* tests; *ns, Non-significant.

Increased expression of *gadD3* was observed for strain N1-227 in the pH control relative to the baseline control (*P* < 0.05, Figure 2). Additionally, acetic acid or lactic acid habituation resulted in significant upregulation of *gadD3* as compared to the pH control in both N1-227 and R2-499 (*P* < 0.05). No significant change of *gadD2* expression was observed for both strains in the pH control compared to the baseline control. However, similar to *gadD3*, acetic acid or lactic acid habituation induced significant and dramatic expression of *gadD2* in comparison with pH control in both strains (*P* < 0.05). The qPCR results for both strains also showed no significant changes in the expression of gene encoding arginine deiminase (*arcA*) in the pH control relative to the baseline control, and that acetic acid or lactic acid habituation significantly increased *arcA* expression in both strains (*P* < 0.05, Figure 2).

In contrast, transcription of genes related to bile tolerance was variable between the strains. Habituation to lactic acid or acetic acid significantly increased *bsh* gene expression in comparison with the pH control for both strains (*P* < 0.05). However, the pH control had no significant effect on *bsh* expression relative to the baseline control in strain N1-227 (Figure 2). Changes in the expression of *bilE* were also strain-dependent. For strain N1-227, *bilE* was significantly overexpressed (*P* < 0.05) when cells were habituated to acetic or L-lactic acid, whereas no significant changes were observed in strain R2-499. Finally, qPCR data showed habituation to L-lactic acid or acetic acid significantly (*P* < 0.05) induced *sigB* expression in strain N1-227 cells compared to the baseline control (Figure 2A). However, no significant change on *sigB* expression was observed between treatments in strain R2-499 (Figure 2B).

### Influence of Acid Habituation on Expression of Virulence Genes

**FIGURE 3:**
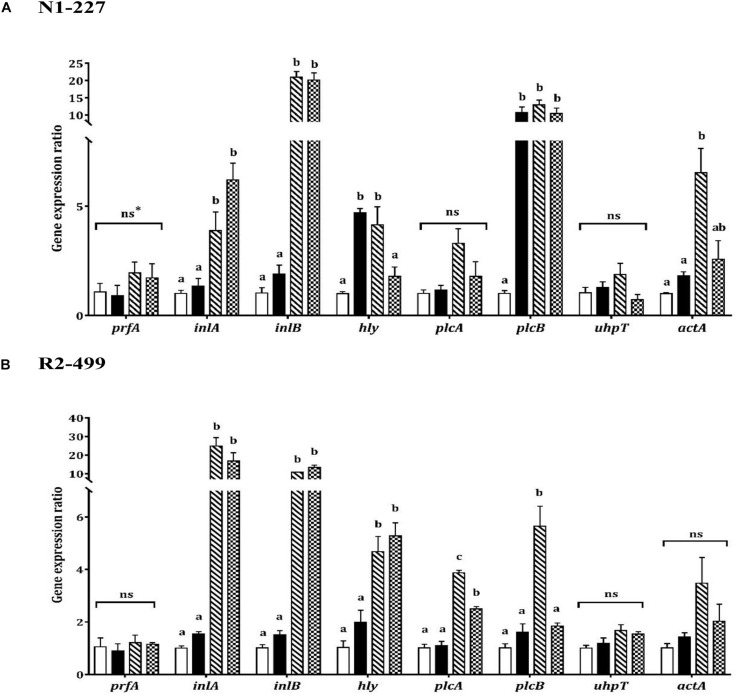
Relative gene expression of virulence related genes in 20 min habituated *Listeria monocytogenes* **(A)** N1-227 and **(B)** R2-499 cells in comparison with non-habituated cells (baseline control, TSB pH 7.4, 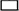). Habituated treatments include as follows: TSB at pH 6.0 (pH control, 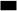), TSB at pH 6.0 with 4.75 mM of acetic acid (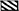) and TSB pH 6.0 with 4.75 mM of L-lactic acid (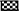). Error bars represent standard error of mean for two biological trails with three replicates for each trail. Different letters indicate that treatments are significantly different (*p* < 0.05) as determined by one-way ANOVA with Tukey’s *post-hoc* tests. *ns, Non-significant.

As was observed with stress genes, qPCR results showed similarities and differences between strains with respect to virulence gene expression in response to organic acid habituation (Figure 3). The transcription level of *prfA* or *uhpT* was not significantly impacted by pH or acid exposure in either strain. However, expression of *inlA, inlB* and *hly* increased in both strains when the pH was decreased. Both strains showed significantly (*P* < 0.05) increased expression of *inlA* and *inlB* in organic acid habituated cells compared to the baseline control or pH control (Figure 3). Furthermore, *hly* expression was significantly (*P* < 0.05) increased in R2-499 cells habituated to acetic acid or L-lactic acid relative to baseline control and pH control. However, significant (*P* < 0.05) overexpression of *hly* in strain N1-227 compared to baseline control was only observed with the pH control and acetic acid habituation treatment.

The qPCR results showed the expression profile for the other virulence genes (*plcA, plcB, actA*) was also strain-dependent (Figure 3). No significant changes were observed in *plcA* expression for strain N1-227 (Figure 3A), while organic acid habituation significantly (*P* < 0.05) increased expression of this gene in strain R2-499 compared to baseline control and pH control (Figure 3B). All three acid treatments (pH control and organic acid habituated cells) significantly (*P* < 0.05) induced *plcB* expression compared to the baseline control in strain N1-227, whereas significant induction in strain R2-499 was only observed with the acetic acid treatment. Conversely, no significant differences were recorded in *actA* expression for strain R2-499, and only acetic acid habituated N1-227 cells showed a significant (*P* < 0.05) increase in the expression level of this gene (Figure 3).

### Effect of Habituation to Organic Acid on *Galleria mellonella* Survivability

The lethal time to 50% population mortality (LT_50_) of each treatment for both strains (Table 3) was determined based on the survival of *G. mellonella* over 5 days post-injection (see Supplementary Figure 1). For both N1-227 and R2-499, the LT_50_ of larvae injected with *L. monocytogenes* habituated with HCl (pH control) was lower than that of larvae injected with non-habituated *L. monocytogenes* (baseline control) and LT_50_ values decreased considerably more when larvae were injected with organic acid habituated *L. monocytogenes* (Table 3). The shortest LT_50_ results were noted with organic acid habituated *L. monocytogenes* R2-499, which suggests this strain may be more virulent than N1-227.

**TABLE 3:** Lethal times until 50% population mortality (LT_50_) for *Galleria mellonella* larva injected with *Listeria monocytogenes* strains habituated to various acid treatments.

**Strain**	**Treatments**	**LT_50_ (Hours) (95% CI)**
**N1-227**	Baseline	40.72 (32.58–50.90)**^a^**
	pH control	34.23 (26.28–44.59)**^a^**
	Acetic acid	19.76 (15.50–25.19)**^b^**
	L-lactic acid	17.14 (13.58–21.65)**^b^**
**R2-499**	Baseline	37.23 (31.22–44.39)**^a^**
	pH control	29.83 (22.79–39.04)**^a^**
	Acetic acid	17.14 (13.10–22.42)**^b^**
	L-lactic acid	14.01 (10.97–17.88)**^b^**

*Different letters within a strain indicate that treatments are significantly different (*p* < 0.05) as determined by Probit analysis.*

To test whether the previous organic acid habituation affected the survival or growth of *L. monocytogenes* in *G. mellonella* larvae, post-injection bacterial cell numbers were determined over time. The number of *L. monocytogenes* cells showed a slight decrease for the first 5 h and then remained constant through the 20 h sampling period (see Supplementary Figure 2). Other researchers have also reported that *L. monocytogenes* cells decreased in number for the first 2 h post-injection (Joyce and Gahan, 2010; Schrama et al., 2013). No statistically significant differences were observed between treatments for either *L. monocytogenes* strain, indicating that the enhanced virulence observed in organic acid habituated cells is not due to enhanced survival or growth in the larvae.

## Discussion

The qPCR experiments showed organic acid habituation impacted the expression of genes encoding important acid and bile stress response mechanisms in both strains of *L. monocytogenes*. The GAD system serves as a key mechanism of *L. monocytogenes* survival in acid environments (Cotter et al., 2001; Melo et al., 2015). Karatzas et al. (2012) proposed a model wherein GAD-mediated acid resistance consists of two semi-independent systems: An intracellular system that involves GadD3 acting on intracellular glutamate and an extracellular system that involves GadD2 decarboxylation of glutamate imported by the antiporter GadT2. Interestingly, the differential induction of *gadD3* vs. *gadD2* in strains N1-227 and R2-499 suggests that *gadD3* may play a more prominent role in acid protecting in N1-227, while *gadD2* serves as primary defense mechanism in R2-499. Additionally, the fold-change in *bilE* expression was lower than that of *bsh* in both strains, which might be a consequence of cell growth phase. Sue et al. (2003) showed that *bilE* expression is growth phase-dependent, with highest expression level observed in stationary phase cells, and this study used cells collected at mid-log phase.

Infection of host cells by *L. monocytogenes* can be divided into three stages that require specific virulence factors: Initial cell invasion (InlA and InlB), escape from vacuole (Hly, PlcA, and PlcB) and cell-to-cell spread (ActA and UhpT) (Cossart et al., 1989; Mélanie et al., 2006; Schnupf and Portnoy, 2007; Joyce and Gahan, 2010; Hamon et al., 2012). It has been reported that *L. monocytogenes* is able to sense different environments and host cell compartments and regulate virulence gene expression accordingly (Freitag and Jacobs, 1999; Gaballa et al., 2019). Other researchers have found that *inlA* and *inlB* are induced prior to the cell invasion, while *hly*, *plcB*, and *plcA* are overexpressed within the phagosome and *uhpT* and *actA* are expressed in the cytosol (Bubert et al., 1999). In this study, *inlA* and *inlB* showed a similar expression pattern in both strains in response to acid exposure (Figure 3). Significant induction of other virulence genes in response to pH or acid was also observed but the patterns were strain-dependent. Additionally, although the transcription level of *prfA* was not significantly altered by acid exposure, *hly* transcription is PrfA-dependent (Kazmierczak et al., 2006; Scortti et al., 2007; de las Heras et al., 2011). The observed induction of *hly* may therefore reflect post-transcriptional control of PrfA activity in these cells.

In summary, RT-qPCR demonstrated that habituation to L-lactic or acetic acids induces statistically significant increases in the expression of several genes associated with acid and bile stress resistance in two *L. monocytogenes* strains that are known human pathogens. While many of these changes were strain-specific, induction patterns for several stress and virulence genes, including *gadD2*, *arcA*, *bsh*, two internalin genes *ilnA* and *ilnB*, in response to acid habituation were similar between N1-227 and R2-499. Future studies might explore the role of nucleotide polymorphism in promoter sequences or in DNA binding motifs in gene expression patterns.

Organic acid habituation also enhanced *in situ* virulence of both *L. monocytogenes* strains as evidenced by a reduced the LT_50_ value in the *in vivo G. mellonella* infection model. Our finding that HCl or organic acid habituation enhanced virulence of both strains in the *G. mellonella* model stands in contrast with the report of Schrama et al. (2013), who observed acid or salt adaptation reduced the infectious ability of some *L. monocytogenes*. However, factors such as different strains and stressors may have contributed to this discrepancy. Our prior research suggests this difference is likely due to strain-specific variation among *L. monocytogenes* (Zhang et al., 2014), and illustrates the need for further study to determine how widespread this phenomenon is among pathogenic and non-pathogenic strains of *L. monocytogenes*.

Taken together, these results suggest that exposure to organic acids can increase the pathogenicity of some *L. monocytogenes* strains by enhancing their ability to survive passage through the gastrointestinal tract while simultaneously priming them for intracellular virulence. While our prior results indicate that this phenomenon may not be universally shared among strains of *L. monocytogenes* (Zhang et al., 2014), the fact that it does occur in pathogenic strains associated with foodborne outbreaks (Table 1) underscores the potential for organic acids to have unanticipated consequences on food safety and public health. To fully understand the broader impact, future studies are needed to determine how widespread this phenomenon is among additional strains of *L. monocytogenes*, including both known human pathogens and strains not currently recognized as pathogenic, and to examine the impact of food systems and conditions encountered during processing and storage such as refrigeration temperatures.

## Data Availability Statement

The original contributions presented in the study are included in the article/Supplementary Material, further inquiries can be directed to the corresponding author/s.

## Author Contributions

CC and JB: conceptualization, data curation, funding, acquisition, project administration, supervision, validation, and writing—review and editing. ML: formal analysis, investigation, methodology, data collection, software, visualization, and writing—original draft. All authors contributed to the article and approved the submitted version.

## Conflict of Interest

The authors declare that the research was conducted in the absence of any commercial or financial relationships that could be construed as a potential conflict of interest.
