# Supplementary material for: Organic Acid Exposure Enhances Virulence in Some Listeria monocytogenes Strains Using the Galleria mellonella Infection Model
Source: Front Microbiol. 2021 Jul 6;12:675241. doi: 10.3389/fmicb.2021.675241 (PMC8290484; doi:10.3389/fmicb.2021.675241)
Supplement: Supplementary file 1 [file Data_Sheet_1.docx]

**N1-227**

**
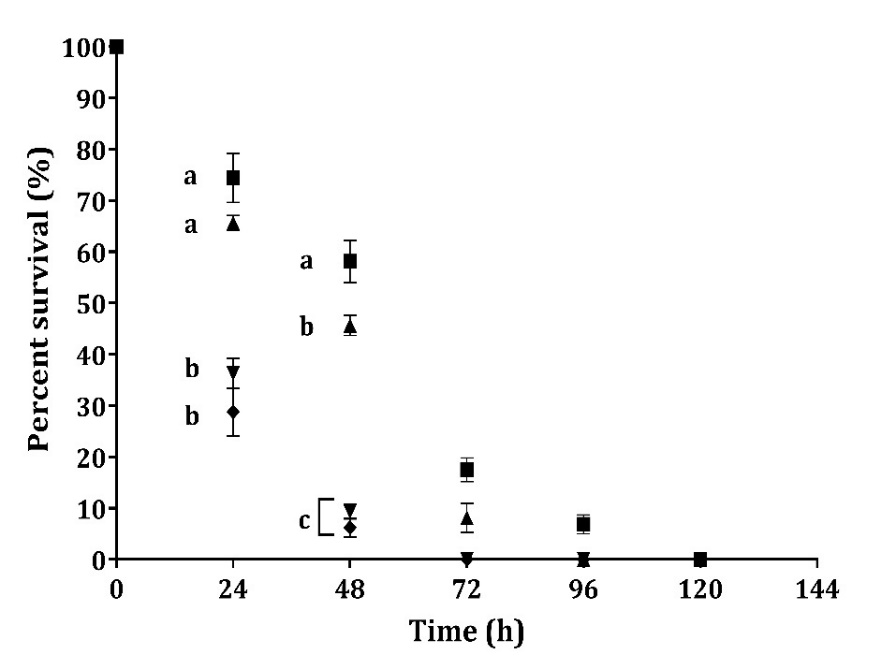
**

**R2-499**

**
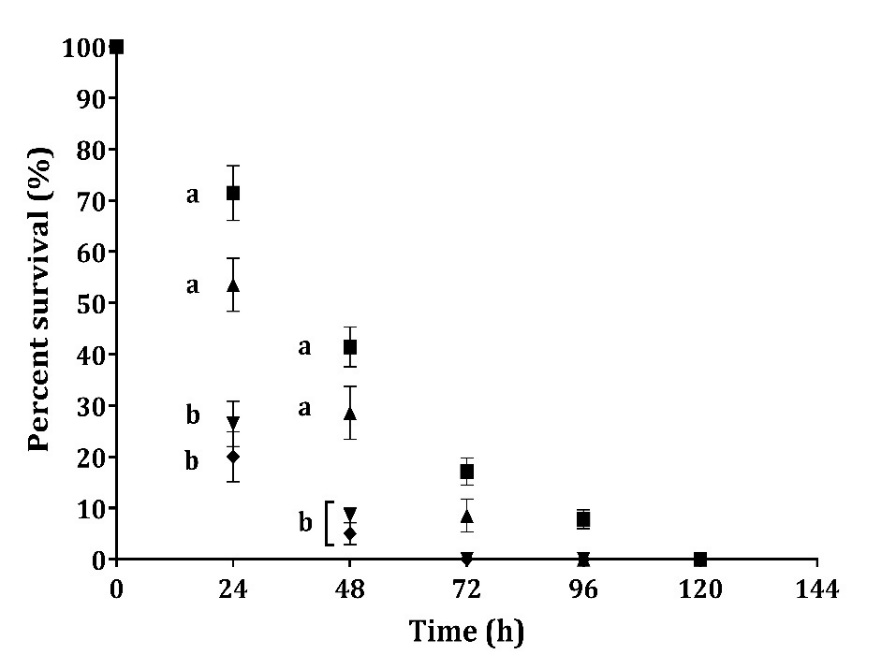
**

**Figure S1** Survival of *Galleria mellonella* larvae after injection with habituated or non-habituated (baseline control, TSB pH 7.4,
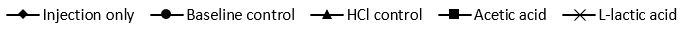
) *Listeria monocytogenes* N1-227 (panel A) and R2-499 (panel B) cells. Media included in the habituated cells are as follows: TSB pH 6.0 (pH control,
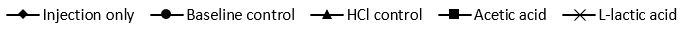
), TSB pH 6.0 w/ 4.75 mM of acetic acid (
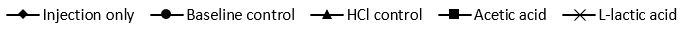
) and TSB pH 6.0 w/ 4.75 mM of L-lactic acid (
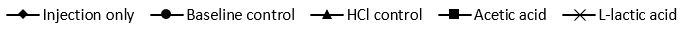
). Error bars represent standard error of mean for two biological trails. Different letters indicate that treatments are significantly different (p<0.05) as determined by one-way ANOVA with Tukey’s post-hoc tests.

**N1-227**

**R2-499**

**Figure S2** Growth of habituated and non-habituated *Listeria monocytogenes* N1-227 (A) and R2-499 (B) cells in *Galleria mellonella* after 5, 10, 15 and 20 h post injection. Media included: TSB pH 7.4 (baseline control,
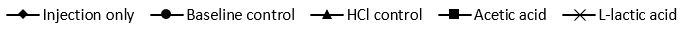
), TSB pH 6.0 (pH control,
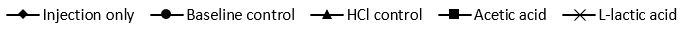
), TSB pH 6.0 w/ 4.75 mM of acetic acid (
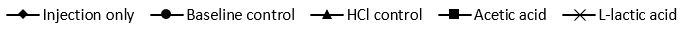
) and TSB pH 6.0 w/ 4.75 mM of L-lactic acid (
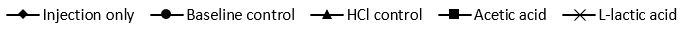
). Error bars represent standard error of mean for two biological trails. No significant differences were present between treatments as determined by one-way ANOVA with Tukey’s post-hoc tests.
